# Comparative outcomes of ceftazidime-avibactam versus meropenem-vaborbactam for KPC-producing Enterobacterales infections

**DOI:** 10.1128/aac.01602-25

**Published:** 2026-03-23

**Authors:** Sara M. Karaba, Dariusz A. Hareza, Jose M. Munita, Jose R. W. Martinez, Yehudit Bergman, Armani M. Hawes, Zackery P. Bulman, Sara E. Cosgrove, Patricia J. Simner, Pranita D. Tamma

**Affiliations:** 1 Department of Medicine, Johns Hopkins University School of Medicine 1500 , Baltimore, Maryland, USA; 2 Instituto de Ciencias e Innovación en Medicina, Facultad de Medicina Clínica Alemana, Universidad del Desarrollo 28071, Santiago, Chile; 3 Department of Pediatrics, Johns Hopkins University School of Medicine 1500 , Baltimore, Maryland, USA; 4 Department of Pharmacy Practice, Retzky College of Pharmacy, University of Illinois Chicago 14681, Chicago, Illinois, USA; 5 Department of Pathology, Johns Hopkins University School of Medicine 1500 , Baltimore, Maryland, USA; University of California San Francisco, San Francisco, California, USA

**Keywords:** carbapenemases, CRE, antimicrobial resistance

## Abstract

Thirty-day mortality was similar in a propensity-score-weighted cohort of 73 patients with KPC-producing Enterobacterales infections treated with ceftazidime-avibactam or meropenem-vaborbactam. Emergence of resistance was higher in the ceftazidime-avibactam group (12% vs 0%); putative resistance mechanisms included porin mutations (*Klebsiella pneumoniae*) and R2 loop structural changes in AmpC (*Enterobacter cloacae* complex).

## INTRODUCTION

Carbapenem-resistant Enterobacterales (CRE) represent a major global health threat. *Klebsiella pneumoniae* carbapenemases (KPCs) are a predominant driver of carbapenem resistance worldwide (1–5). Among clinical CRE isolates from across the United States in 2019 to 2021, 83% were carbapenemase-producing, with approximately 80% harboring *bla*_KPC_ genes (6). The global spread of *bla*_KPC_ has been largely facilitated by the dissemination of *K. pneumoniae* clonal group 258, with the *bla*_KPC_ gene typically embedded within the TN4401 transposon, enhancing mobility (5, 7–9). More recent reports suggest widespread transmission of *bla*_KPC_ across diverse bacterial species, lineages, plasmid types, and transposon locations, highlighting a growing challenge in containment and treatment (10–13).

In the United States, KPC-producing Enterobacterales infections are primarily treated with ceftazidime-avibactam (CZA) or meropenem-vaborbactam (MVB) (14). The lack of clinical trials comparing these agents makes it difficult for clinicians to preferentially select between them. Observational data suggest that 30-day mortality is approximately 24% for patients receiving CZA and 18% for those treated with MVB (15–20). The emergence of resistance has been observed in approximately 13% (CZA-treated) and 8% (MVB-treated) of cases (15–19).

Although the combined endpoints of mortality and resistance emergence seem to favor MVB, the available data have limitations. Most studies derive outcome estimates from independent cohorts treated with either CZA or MVB, preventing direct comparisons within the same patient population (15–18). In one study directly comparing CZA and MVB within the same cohort, carbapenemase testing was performed for only 34% of isolates (19). Even when comparators are available, observational studies are prone to confounding by indication—sicker or more complex patients may be more likely to receive one agent over the other. Furthermore, several studies did not confirm CZA or MVB susceptibility using reference broth microdilution (BMD), raising concerns about potential misclassification (17–20).

To address several of these limitations, we aimed to expand the existing literature by comparing the odds of mortality and resistance emergence in patients treated with CZA or MVB for KPC-producing Enterobacterales infections.

**Setting and participants**: We conducted a prospective observational study of unique, consecutive patients with KPC-producing Enterobacterales infections who received CZA or MVB for at least 72 h between January 2020 and December 2023 at three Johns Hopkins Health System hospitals. Eligible patients had an isolate resistant to at least one carbapenem but susceptible to the administered treatment (CZA or MVB) based on BMD results. Patients with infections caused by organisms harboring *bla*_NDM_, *bla*_VIM_, *bla*_IMP_, or *bla*_OXA-48-like_ genes were excluded. Day 1 was defined as the date of collection of the first positive culture.

**Outcomes**: The primary outcome was 30-day mortality. For patients with a recurrent clinical culture growing the same KPC-producing species within 90 days of the index infection, development of resistance to the antibiotic prescribed (i.e., CZA or MVB) was explored. The development of resistance was defined as a change from susceptible to resistant comparing the index and subsequent isolate, applying Clinical and Laboratory Standards Institute (CLSI) criteria (21).

**Data extraction**: The following data were entered into a REDCap database, with agreement by at least two infectious diseases physicians (S.M.K., D.A.H., P.D.T.): demographics, pre-existing medical conditions, severity of illness, source of infection and source control measures, bacterial species, genetic resistance markers, minimum inhibitory concentration (MIC) data, antibiotic treatment, and outcomes. Baseline data were collected on day 1 of illness. The study was approved by the Institutional Review Board at Johns Hopkins University with a waiver of informed consent.

**Microbiologic and antimicrobial susceptibility testing (AST)**: After routine clinical microbiology evaluations for species identification and AST, isolates were stored at −80°C. Isolates were sub-cultured from frozen stock onto tryptic soy broth agar. Species identification was confirmed using MALDI TOF MS. MICs for CZA and MVB (both for index and subsequent isolates) were determined using custom frozen reference BMD panels (22). Susceptibility was assessed according to CLSI criteria (21).

**Whole genome sequencing (WGS)**: The Illumina MiSeq platform was used to construct assemblies to confirm the presence of *bla*_KPC_ genes and to determine putative mechanisms of resistance in subsequent isolates that developed resistance to the treatment agent received. Further information on WGS methods and analysis is described in the Supplemental methods.

**Statistical analysis**: Baseline data were compared using Fisher’s exact test for categorical variables and the Wilcoxon rank sum test for continuous variables. Inverse probability weighting (IPW) with propensity scores was applied to ensure comparability between the exposed (CZA) and unexposed (MVB) groups. Variables used to generate propensity scores were determined *a priori* and are described in Table 1. Patients in the CZA group were weighted by the inverse of their propensity score, while those in the MVB group were weighted by one minus the inverse of their propensity score, creating a pseudo-population for outcome analysis. Balance between groups was assessed using standardized mean differences (SMDs), with absolute SMDs between −0.20 and 0.20 indicative of adequate balance (23). The odds ratio (OR) for 30-day mortality was estimated using logistic regression via the “kmatch ipw” function in Stata (version 17.0, StataCorp, College Station, TX). A two-sided *P*-value <0.05 was considered statistically significant.

**TABLE 1:** Baseline characteristics of 73 patients treated with ceftazidime-avibactam or meropenem-vaborbactam for Enterobacterales infections harboring *bla*_KPC_ genes

Characteristics		Full cohort			Inverse probability weighted cohort^*a*^		
	Total (*N* = 73)	Ceftazidime-avibactam (57, 78.1%)	Meropenem-vaborbactam (16, 21.9%)	*P*-value	Ceftazidime-avibactam (%)	Meropenem-vaborbactam (%)	Standardized mean difference
Age ≥ 65 years	34 (46.6%)	28 (49.1%)	6 (37.5%)	0.57	46.78	47.29	−0.0080617
Median age [IQR]	64 [56–72]	64 [57–72]	56 [35–69]	0.13			
Female sex	30 (41.1%)	21 (36.8%)	9 (56.3%)	0.25			
Severity of illness							
Intensive care unit admission	51 (69.9%)	38 (66.7%)	13 (81.3%)	0.36	69.39	71.62	−0.0522873
Vasopressors	43 (58.9%)	35 (61.4%)	8 (50.0%)	0.57			
Mechanical ventilation	38 (52.1%)	27 (47.4%)	11 (68.8%)	0.16			
Severe immunocompromise^*b*^	11 (15.1%)	9 (15.8%)	2 (12.5%)	1.0	15.52	18.30	−0.0747875
Charlson Comorbidity Index ≥5	47 (64.4%)	38 (66.7%)	9 (56.3%)	0.56	64.98	71.75	−0.1318941
Renal replacement therapy	26 (35.6%)	21 (36.8%)	5 (31.3%)	0.77	35.82	40.13	−0.0860912
Site of Infection							
Intra-abdominal	5 (6.9%)	4 (7.0%)	1 (6.3%)	1.0			
Bacteremia	22 (30.1%)	17 (29.8%)	5 (31.3%)	1.0			
Pneumonia	21 (28.8%)	18 (31.6%)	3 (18.8%)	0.37			
Skin or soft tissue	15 (20.6%)	9 (15.8%)	6 (37.5%)	0.08			
Urine	9 (12.3%)	9 (15.8%)	0 (0%)	0.19			
Uncontrolled source of infection by day 14^*c*^	16 (21.9%)	11 (19.3%)	5 (31.3%)	0.32	20.03	15.42	0.01972139
*Escherichia coli*	4 (5.5%)	3 (5.3%)	1 (6.3%)	1.0	5.45	3.70	0.0708201
*Klebsiella pneumoniae*	43 (58.9%)	35 (61.4%)	8 (50.0%)	0.57	59.82	62.70	−0.0528657
*Enterobacter cloacae* complex	10 (13.7%)	8 (14.0%)	2 (12.5%)	1.0	14.03	16.79	−0.077343

^*a*^ Variables listed in the columns below were incorporated in the generation of propensity scores.

^*b*^ The following categories comprised severe immunocompromise: bone marrow transplant in the last 12 months (0, 0%), active graft versus host disease on immunosuppressive therapy (1, 1.4%), chemotherapy in the last 6 months (3, 4%), solid organ transplant (6, 8%), AIDS (0, 0%), autoimmune condition requiring corticosteroids or biologics (1, 1.4%), or an absolute neutrophil count <500 cells/microliter between days 1 to 7 (2, 3%); categories are not mutually exclusive.

^*c*^ Persistent sources of infection included undrained abscesses or indwelling catheters not removed while receiving therapy with either ceftazidime-avibactam or meropenem-vaborbactam.

Among 73 eligible patients, 57 (78%) received CZA (2.5 g every 8 h), and 16 (22%) received MVB (4 g every 8 h), both infused over 3 h, with renal dose adjustments as appropriate. The median duration of therapy was 15 days in both groups [CZA IQR 8–20 vs MVB IQR 9–21]. Baseline characteristics for the overall cohort and the CZA and MVB subgroups are summarized in Table 1. Seventy percent of patients were in the ICU at the time of culture collection. Common bacterial species were *K. pneumoniae* (59%), *Enterobacter cloacae* complex (14%), and *Escherichia coli* (6%). Of the 73 isolates, 35% and 65% contained *bla*_KPC-2_ and *bla*_KPC-3_ genes, respectively. Baseline characteristics were similar between the two groups. After IPW, balance was achieved across all variables, with all SMDs between −0.20 and 0.20.

Thirty-day mortality was 14% (8/57) and 19% (3/16) in the CZA and MVB groups, respectively (*P* = 0.70). In the IPW cohort, there was no difference in the odds of death by day 30 between the two treatment groups (aOR = 1.04, 95% CI 0.87–1.25, *P* = 0.66). Twenty-four patients (33%) experienced a recurrent infection from any site with a KPC-producing isolate of the same species within 90 days of the initial positive culture (Table S1). Of these, 17/57 (30%) and 7/16 (44%) were in the CZA and MVB groups, respectively (*P* = 0.37). Emergence of resistance occurred in 12% (2/17) and 0% (0/7) for patients receiving CZA or MVB for the index infection, respectively.

Regarding the two isolates for which CZA resistance emerged, one was a ST258 *K. pneumoniae* and the second was a ST171 *E. cloacae* complex. Both isolates contained *bla*_KPC-3_ genes, which were harbored on a ΔTn1331-Tn4401d nested transposon element. Neither isolate harbored additional acquired β-lactamase genes or mutations in the *bla*_KPC_ gene. In the *K. pneumoniae* strain, the increase in CZA MIC from 0.5 to 32 µg/mL was potentially due to the combination of porin deletions in OmpK36 (ΔM1-A22) and OmpK37 (ΔF384). In the *E. cloacae* strain, the increased CZA MIC from 8 to 64 µg/mL was potentially associated with a deletion of Ala294–Pro295 in AmpC.

In a cohort of 73 patients with invasive KPC-producing Enterobacterales infections, approximately 15% died within 30 days, a proportion consistent with previously published reports (15–19). Within the IPW cohort, 30-day mortality was similar between patients receiving CZA or MVB. To date, no clinical trials have directly compared CZA and MVB for KPC-producing infections. Although limited by the small sample size, our analysis identified a potential difference in the emergence of resistance: resistance developed in 12% of patients treated with CZA, whereas no resistance was observed among patients receiving MVB. Notably, resistance estimates were derived only from patients with recurrent infections and may, therefore, underestimate the true incidence that might have been detected through systematic post-treatment surveillance cultures. Nonetheless, these findings are broadly consistent with prior studies reporting emergence of resistance ranging from 3% to 32% for CZA and from 0% to 17% for MVB (15–19).

Previous studies suggest that dose reductions of newer β-lactams in patients receiving renal replacement therapy can lead to failure in achieving pharmacokinetic/pharmacodynamic targets, ultimately leading to poor patient outcomes including subsequent resistance (15, 24). One of two patients in our study whose isolates developed CZA resistance required continuous renal replacement therapy and underwent CZA dosage reductions. The possibility of suboptimal outcomes with CZA dosage reductions in patients requiring renal replacement needs to be investigated further.

Key mechanisms of Enterobacterales resistance to CZA include (i) acquisition or upregulation of β-lactamases not inhibited by avibactam (e.g., metallo-β-lactamases); (ii) structural alterations in serine β-lactamases (e.g., KPC, OXA-48, AmpC) reducing avibactam binding or enhancing ceftazidime hydrolysis; (iii) increased expression or copy number of β-lactamase genes; (iv) decreased outer membrane permeability due to porin loss or modification; and (v) upregulation of efflux pumps (25). While our findings remain putative and lack experimental validation, resistance in the *K. pneumoniae* isolate may be attributable to deletions in outer membrane proteins OmpK36 and OmpK37, which can diminish β-lactam permeability (26, 27). In contrast, resistance in the *E. cloacae* isolate appears to involve structural modifications in the AmpC R2 loop, a mechanism previously linked to enhanced ceftazidime hydrolysis and reduced avibactam inhibition (28, 29).

This study is observational, retrospective, limited in size and geography, and subject to residual confounding despite inclusion of propensity score weighting. However, our findings underscore an important consideration: while survival for KPC-producing Enterobacterales infections appears comparable between CZA and MVB, CZA may be more prone to the emergence of resistance. Larger studies with geographically diverse isolates are needed to validate our findings as these data are important for guiding antibiotic selection and hospital formulary decisions.

## SUPPLEMENTAL MATERIAL

Supplemental material aac.01602-25-s0001.docx Supplemental methods.

Table S1 aac.01602-25-s0002.docx Antibiotic susceptibility testing results from broth microdilution for patients with recurrent KPC-producing Enterobacterales infections initially treated with CZA or MVB with subsequent recurrent infections with the same bacterial species within 90 days.

ASM does not own the copyrights to Supplemental Material that may be linked to, or accessed through, an article. The authors have granted ASM a non-exclusive, world-wide license to publish the Supplemental Material files. Please contact the corresponding author directly for reuse.
