# Supplementary material for: Comparative outcomes of ceftazidime-avibactam versus meropenem-vaborbactam for KPC-producing Enterobacterales infections
Source: Antimicrob Agents Chemother. 2026 Mar 23;70(5):e01602-25. doi: 10.1128/aac.01602-25 (PMC13148039; doi:10.1128/aac.01602-25)
Supplement: Supplemental material — Supplemental methods. [file aac.01602-25-s0001.docx]

**Supplemental Methods**

**Whole Genome Sequencing and Bioinformatic Analysis.** Whole genome sequencing (WGS) was performed on *Klebsiella pneumoniae* and *Enterobacter cloacae* clinical isolates, both harboring *bla*_KPC-3_, in which resistance to ceftazidime-avibactam (CZA) emerged within 90 days of treatment. The respective index isolates were susceptible to CZA. For downstream comparative analyses, reference genomes for *K. pneumoniae* (ATCC® 13883) and *E. cloacae* (ATCC® 13047) were selected.

Genomic DNA was extracted and sequenced on the Illumina MiSeq platform, generating 150 bp paired-end reads with a target coverage depth exceeding 30x. Raw reads quality was determined by FASTQC v0.11.9 and MultiQC v1.10.1(1). The reads were paired and trimmed with Trimmomatic v0.39 after excluding reads with a quality score on a Phred scale below 30 (2). Genomes were assembled *de novo* using SPAdes v3.14.0 (3). The quality of the assemblies was assessed with QUAST v5.0 (4). Assembled contigs were screened for acquired antimicrobial resistance determinants using ResFinder v4.1 and AMRFinder applying thresholds of ≥90% nucleotide identity and ≥60% gene coverage (5,6). Mutations previously implicated in CZA resistance were identified via PointFinder and manual curation following a review of the published literature. Genome annotation was performed with Prokka, and multilocus sequence types were assigned using the MLST tool (7).

In *K. pneumoniae*, the primary resistance mechanisms investigated included mutations in *bla*_KPC_, particularly within the Ω-loop (e.g., D179Y), increased *bla*_KPC_ copy number, and disruptions in outer membrane porins (i.e., OmpK35, OmpK36, OmpK37). For *E. cloacae*, the most well-characterized resistance mechanism examined was deletion or alteration of the R2 loop in the chromosomal AmpC β-lactamase (e.g., A294_P295del), which has been associated with enhanced ceftazidime hydrolysis and reduced susceptibility to avibactam inhibition. Additional mechanisms assessed included loss of porins (OmpC and OmpF) and upregulation of the AcrAB-TolC efflux system.

**References**

1. Ewels P, Magnusson M, Lundin S, Käller M. MultiQC: summarize analysis results for multiple tools and samples in a single report. Bioinformatics [Internet]. 2016 Oct 1 [cited 2021 Nov 9];32(19):3047–8.

2. Bolger AM, Lohse M, Usadel B. Trimmomatic: a flexible trimmer for Illumina sequence data. Bioinformatics [Internet]. 2014 Aug 1 [cited 2021 Nov 9];30(15):2114–20. Available from: https://academic.oup.com/bioinformatics/article/30/15/2114/2390096

3. Bankevich A, Nurk S, Antipov D, Gurevich AA, Dvorkin M, Kulikov AS, et al. SPAdes: A new genome assembly algorithm and its applications to single-cell sequencing. Journal of Computational Biology. 2012 May 1;19(5):455–77.

4. Gurevich A, Saveliev V, Vyahhi N, Tesler G. QUAST: Quality assessment tool for genome assemblies. Bioinformatics [Internet]. 2013 Apr 15 [cited 2021 Apr 13];29(8):1072–5. Available from: https://pubmed.ncbi.nlm.nih.gov/23422339/

5. Feldgarden M, Brover V, Gonzalez-Escalona N, Frye JG, Haendiges J, Haft DH, et al. AMRFinderPlus and the Reference Gene Catalog facilitate examination of the genomic links among antimicrobial resistance, stress response, and virulence. Scientific Reports 2021 11:1 [Internet]. 2021 Jun 16 [cited 2024 May 30];11(1):1–9. Available from: https://www.nature.com/articles/s41598-021-91456-0

6. Feldgarden M, Brover V, Haft DH, Prasad AB, Slotta DJ, Tolstoy I, et al. Validating the AMRFINder tool and resistance gene database by using antimicrobial resistance genotype-phenotype correlations in a collection of isolates. Antimicrob Agents Chemother. 2019;63(11).

7. Seemann T. Prokka: rapid prokaryotic genome annotation. Bioinformatics [Internet]. 2014 Jul 15 [cited 2019 Oct 20];30(14):2068–9.
