# Supplementary material for: Comparative outcomes of ceftazidime-avibactam versus meropenem-vaborbactam for KPC-producing Enterobacterales infections
Source: Antimicrob Agents Chemother. 2026 Mar 23;70(5):e01602-25. doi: 10.1128/aac.01602-25 (PMC13148039; doi:10.1128/aac.01602-25)
Supplement: Table S1 — Antibiotic susceptibility testing results from broth microdilution for patients with recurrent KPC-producing Enterobacterales infections initially treated with CZA or MVB with subsequent recurrent infections with the same bacterial species within 90 days. [file aac.01602-25-s0002.docx]

**Supplemental Table 1:** Antibiotic susceptibility testing results from broth microdilution for patients with recurrent KPC-producing Enterobacterales infections initially treated with ceftazidime-avibactam (CZA) or meropenem-vaborbactam (MVB) with subsequent recurrent infections with the same bacterial species within 90 days

| **Patient number** | **Organism** | **Antibiotic administered^1^** | **Index CZA MIC (µg/mL)** | **Subsequent CZA MIC (µg/mL)** | **Index MVB MIC (µg/mL)** | **Subsequent MVB MIC (µg/mL)** | **Days between index and subsequent culture** | **Subsequent MIC in the non-susceptible range to the antibiotic administered^2^** |
| --- | --- | --- | --- | --- | --- | --- | --- | --- |
| 1 | *Citrobacter freundii* | CZA | 0.5 | 0.25 | 0.03 | 0.5 | 10 | No |
| 2 | *Klebsiella pneumoniae* | CZA | 1 | 2 | 0.12 | 0.5 | 10 | No |
| 3 | *Klebsiella oxytoca* | CZA | 2 | 8 | 0.03 | 0.06 | 33 | No |
| 4 | *Klebsiella oxytoca* | CZA | 4 | 4 | 0.25 | 0.5 | 17 | No |
| 5 | *Klebsiella pneumoniae* | CZA | 1 | 2 | 0.5 | 1 | 47 | No |
| 6 | *Klebsiella pneumoniae* | CZA | 1 | 2 | 0.03 | 0.06 | 25 | No |
| 7 | *Enterobacter cloacae* complex | CZA | 8 | 64 | 0.03 | 0.03 | 51 | **Yes^3^** |
| 8 | *Klebsiella pneumoniae* | CZA | 0.12 | .25 | 0.015 | 0.25 | 23 | No |
| 9 | *Klebsiella pneumoniae* | CZA | 2 | 4 | 0.03 | 0.5 | 32 | No |
| 10 | *Klebsiella pneumoniae* | CZA | 2 | 2 | 0.06 | 0.5 | 83 | No |
| 11 | *Klebsiella pneumoniae* | CZA | 0.5 | 2 | 0.25 | 0.25 | 41 | No |
| 12 | *Klebsiella pneumoniae* | CZA | 0.25 | 0.5 | 0.03 | 0.06 | 32 | No |
| 13 | *Klebsiella pneumoniae* | CZA | 2 | 2 | 0.03 | 0.06 | 49 | No |
| 14 | *Klebsiella pneumoniae* | CZA | 0.5 | 32 | 0.015 | 0.03 | 11 | **Yes^4^** |
| 15 | *Klebsiella aerogenes* | CZA | 2 | 4 | 2 | 2 | 83 | No |
| 16 | *Klebsiella pneumoniae* | CZA | 1 | 8 | 0.03 | 0.5 | 25 | No |
| 17 | *Klebsiella pneumoniae* | CZA | 2 | 8 | 0.12 | 0.5 | 27 | No |
| 18 | *Citrobacter amalonaticus* | MVB | 0.25 | 0.5 | 0.03 | 0.015 | 26 | No |
| 19 | *Serratia marcescens* | MVB | 0.12 | 0.25 | 0.03 | 0.015 | 55 | No |
| 20 | *Citrobacter freundii* | MVB | 0.12 | 0.25 | 0.03 | 0.03 | 15 | No |
| 21 | *Klebsiella pneumoniae* | MVB | 0.5 | 0.5 | 0.5 | 0.03 | 53 | No |
| 22 | *Klebsiella pneumoniae* | MVB | 2 | 4 | 0.03 | 0.12 | 64 | No |
| 23 | *Klebsiella pneumoniae* | MVB | 2 | 2 | 0.03 | 0.06 | 20 | No |
| 24 | *Klebsiella pneumoniae* | MVB | 2 | 2 | 0.03 | 0.03 | 37 | No |

^1^CZA: ceftazidime-avibactam, MVB: meropenem-vaborbactam; ^2^Clinical and Laboratory Standards Institute susceptibility criteria as follows: CZA: 8/4 µg/mL (susceptible), 16/4 µg/mL (resistant); MVB: 4/8 µg/mL (susceptible), 8/8 µg/mL (intermediate), 16/8 µg/mL (resistant); ^3^Patient did not receive renal replacement therapy; ^4^Patient received renal replacement therapy.
